# A higher CD34 + cell dose correlates with better event-free survival after KIR-ligand mismatched cord blood transplantation for childhood acute myeloid leukemia

**DOI:** 10.1186/s13045-024-01548-3

**Published:** 2024-04-29

**Authors:** Hisashi Ishida, Yuta Kawahara, Daisuke Tomizawa, Yasuhiro Okamoto, Asahito Hama, Yuko Cho, Katsuyoshi Koh, Yuhki Koga, Nao Yoshida, Maho Sato, Kiminori Terui, Naoyuki Miyagawa, Akihiro Watanabe, Junko Takita, Ryoji Kobayashi, Masaki Yamamoto, Kenichiro Watanabe, Keiko Okada, Koji Kato, Kimikazu Matsumoto, Moeko Hino, Ken Tabuchi, Hirotoshi Sakaguchi

**Affiliations:** 1 Department of Pediatrics, Okayama University Hospital, 2-5-1, Shikata-cho, Kita-ku, Okayama city, 700-8558 Okayama Japan; 2 Department of Pediatrics, Jichi Medical University School of Medicine, Shimotsuke, Japan; 3 Children’s Cancer Center, National Center for Child Health and Development, Tokyo, Japan; 4 Department of Pediatrics, Graduate School of Medical and Dental Sciences, Kagoshima University, Kagoshima, Japan; 5 Department of Hematology and Oncology, Children’s Medical Center, Japanese Red Cross Aichi Medical Center Nagoya First Hospital, Nagoya, Japan; 6 Department of Pediatrics, Hokkaido University Hospital, Sapporo, Japan; 7 Department of Hematology/Oncology, Saitama Children’s Medical Center, Saitama, Japan; 8 Department of Perinatal and Pediatric Medicine, Graduate School of Medical Sciences, Kyushu University, Fukuoka, Japan; 9 Department of Hematology/Oncology, Osaka Women’s and Children’s Hospital, Izumi, Japan; 10 Department of Pediatrics, Hirosaki University Hospital, Hirosaki, Japan; 11 Division of Hematology/Oncology, Kanagawa Children’s Medical Center, Yokohama, Japan; 12 Department of Pediatrics, Niigata Cancer Center Hospital, Niigata, Japan; 13 Department of Pediatrics, Kyoto University Hospital, Kyoto, Japan; 14 Department of Hematology/Oncology for Children and Adolescents, Sapporo Hokuyu Hospital, Sapporo, Japan; 15 Department of Pediatrics, Sapporo Medical University Hospital, Sapporo, Japan; 16 Department of Hematology and Oncology, Shizuoka Children’s Hospital, Shizuoka, Japan; 17 Department of Pediatric Hematology/Oncology, Osaka City General Hospital, Osaka, Japan; 18 Central Japan Cord Blood Bank, Seto, Japan; 19 Department of Pediatrics, Chiba University School of Medicine, Chiba, Japan; 20 Japanese Data Center for Hematopoietic Cell Transplantation, Nagakute, Japan

**Keywords:** Acute myeloid leukemia, Children, Cord blood cell transplantation, KIR-ligand

## Abstract

Although killer Ig-like receptor ligands (KIR-L) mismatch has been associated with alloreactive natural killer cell activity and potent graft-versus-leukemia (GVL) effect among adults with acute myeloid leukemia (AML), its role among children with AML receiving cord blood transplantation (CBT) has not been determined. We conducted a retrospective study using a nationwide registry of the Japanese Society for Transplantation and Cellular Therapy. Patients who were diagnosed with *de novo* non-M3 AML and who underwent their first CBT in remission between 2000 and 2021 at under 16 years old were included. A total of 299 patients were included; 238 patients were in the KIR-L match group, and 61 patients were in the KIR-L mismatch group. The cumulative incidence rates of neutrophil recovery, platelet engraftment, and acute/chronic graft-versus-host disease did not differ significantly between the groups. The 5-year event-free survival (EFS) rate was 69.8% in the KIR-L match group and 74.0% in the KIR-L mismatch group (*p* = 0.490). Stratification by CD34 + cell dose into four groups revealed a significant correlation between CD34 + cell dose and EFS in the KIR-L mismatch group (*p* = 0.006) but not in the KIR-L match group (*p* = 0.325). According to our multivariate analysis, KIR-L mismatch with a high CD34 + cell dose (≥ median dose) was identified as an independent favorable prognostic factor for EFS (hazard ratio = 0.19, *p* = 0.029) and for the cumulative incidence of relapse (hazard ratio = 0.09, *p* = 0.021). Our results suggested that higher CD34 + cell doses are crucial for achieving a potent GVL effect in the context of KIR-L-mismatched CBT.

**Supplementary Information:**

The online version contains supplementary material available at 10.1186/s13045-024-01548-3.

## To the Editor

In children with acute myeloid leukemia (AML), hematopoietic stem cell transplantation (HSCT) is an essential treatment modality [1], and cord blood transplantation (CBT) is a well-established procedure [2–4]. Although killer Ig-like receptor ligands (KIR-L) mismatch has been associated with alloreactive natural killer (NK) cell activity and potent graft-versus-leukemia (GVL) effect among adults with AML [5, 6], its roles among children with AML receiving CBT has not been determined [7, 8].

We conducted a retrospective study using a nationwide registry in Japan, and explored the associations of KIR-L incompatibility and other clinical factors with patient outcomes in children with AML who received CBT in complete remission. A detailed description of methods can be found in Additional file 1.

## Findings

A total of 299 patients were included, consisting of 238 patients in the KIR-L match group and 61 patients in the KIR-L mismatch group (Additional file 1: Figure S1). The background characteristics of two groups were overall similar (Additional file 1: Table S1). The median follow-up period for survivors was 7.2 years (range, 0.1–22.4). The cumulative incidence rates of neutrophil recovery, platelet engraftment, and acute/chronic graft-versus-host disease did not differ significantly between the groups (Additional file 1: Figure S2-4).

The 5-year event-free survival (5y-EFS) rate was 69.8% for the KIR-L match group and 74.0% for the KIR-L mismatch group (*p* = 0.490; Table 1). The 5-year cumulative incidences of relapse were 22.3% and 15.2% (*p* = 0.257), and the 5-year cumulative incidences of non-relapse mortality (NRM) were 7.9% and 10.8% (*p* = 0.605) for the KIR-L match and mismatch groups, respectively, and the causes of death were similar between the groups (Additional file 1: Tables S2 and S3).

**Table 1:** Univariate and multivariate analysis for event-free survival

			Univariate analysis		Multivariate analysis	
		*n*	5y EFS (95% CI)	*P* value	Hazard ratio (95% CI)	*P* value
Age at HSCT, years old	0–4	144	73.3 (65.1–79.9)	0.918	ref	
	5–9	78	68.2 (55.9–77.7)		0.83 (0.44–1.57)	0.575
	10–15	77	68.2 (56.0–77.6)		0.77 (0.38–1.55)	0.466
TNC*	< median	147	69.8 (61.4–76.8)	0.772		
	≥ median	147	71.2 (62.9–78.1)			
CD34 + cells*	< median	143	65.1 (56.3–72.6)	0.093		
	≥ median	143	76.3 (68.1–82.7)			
KIR-L	match	238	69.8 (63.2–75.4)	0.490		
	mismatch	61	74.0 (60.4–83.5)			
KIR-L and CD34	KIR-L match-CD34 low	112	67.0 (57.1–75.2)	0.096	ref	
	KIR-L mismatch-CD34 low	31	58.0 (37.9–73.7)		1.22 (0.59–2.50)	0.590
	KIR-L match-CD34 high	115	73.4 (63.9–80.7)		0.77 (0.44–1.35)	0.355
	KIR-L mismatch-CD34 high	28	89.1 (70.0–96.4)		0.19 (0.04–0.85)	0.029
CR status at HSCT	CR1	212	73.4 (66.6–79.0)	0.255	ref	
	CR2	87	63.9 (52.5–73.3)		1.35 (0.81–2.23)	0.249
HSCT Year	2000–2009	97	66.6 (56.2–75.1)	0.393	ref	
	2010–2021	202	72.4 (65.2–78.3)		1.48 (0.86–2.56)	0.158
HCT-CI	0	219	72.9 (66.2–78.5)	0.666		
	1	20	56.2 (29.2–76.4)			
	2	3	66.7 (5.4–94.5)			
	3	1	NA			
	6	1	NA			
Conditioning regimen	chemo-MAC	120	79.3 (70.7–85.6)	0.004	ref	
	TBI-MAC	129	58.6 (49.3–66.8)		1.99 (1.13–3.50)	0.017
	RIC	50	82.4 (67.7–90.9)		0.65 (0.26–1.60)	0.350
GVHD prophylaxis	CSA-based	86	67.5 (56.2–76.4)	0.347		
	TAC-based	208	71.8 (64.8–77.6)			
ATG	No	288	70.6 (64.8–75.7)	0.957		
	Yes	11	70.0 (32.9–89.2)			
ECOG PS	0–1	262	71.1 (65.0–76.4)	0.954		
	2–4	13	75.0 (40.8–91.2)			
Recipient CMV serostatus	Negative	95	72.9 (62.2–81.1)	0.877	ref	
	Positive	175	70.3 (62.7–76.7)		0.99 (0.61–1.62)	0.978
Donor recipient sex mismatch	Match	115	74.3 (64.6–81.7)	0.631		
	F to M	75	67.1 (55.0–76.6)			
	M to F	65	68.1 (54.4–78.5)			
Cytogenetic risk	Favorable	51	74.0 (59.4–84.0)	0.639	ref	
	Intermediate	192	70.0 (62.7–76.2)		1.34 (0.66–2.70)	0.414
	Adverse	56	69.7 (55.4–80.3)		1.48 (0.65–3.35)	0.349
grade II–IV acute GVHD**	No		[-]		ref	
	Yes		[-]		1.10 (0.68–1.78)	0.705

*The median total nucleated cell and CD34 + cell doses were 6.7 × 10^7^/kg (range, 0.01–12.3) and 1.9 × 10^5^/kg (range, 0.01–59.4), respectively

**GVHD was treated as a time-dependent covariate in the multivariate analysis

Abbreviations: EFS, event-free survival; CI, confidence interval; HSCT, hematopoietic stem cell transplantation; TNC, total nucleated cell count; CR, complete remission; KIR, killer cell immunoglobulin-like receptor; HCT-CI, hematopoietic cell transplantation-specific comorbidity index; TBI, total body irradiation; MAC, myeloablative conditioning; RIC, reduced-intensity conditioning; CSA, cyclosporine A; TAC, tacrolimus; ATG, antithymocyte globulin; ECOG, Eastern Cooperative Oncology Group; PS, performance status; CMV, cytomegalovirus; F, female; M, male; GVHD, graft-versus-host disease.

**Fig. 1:**
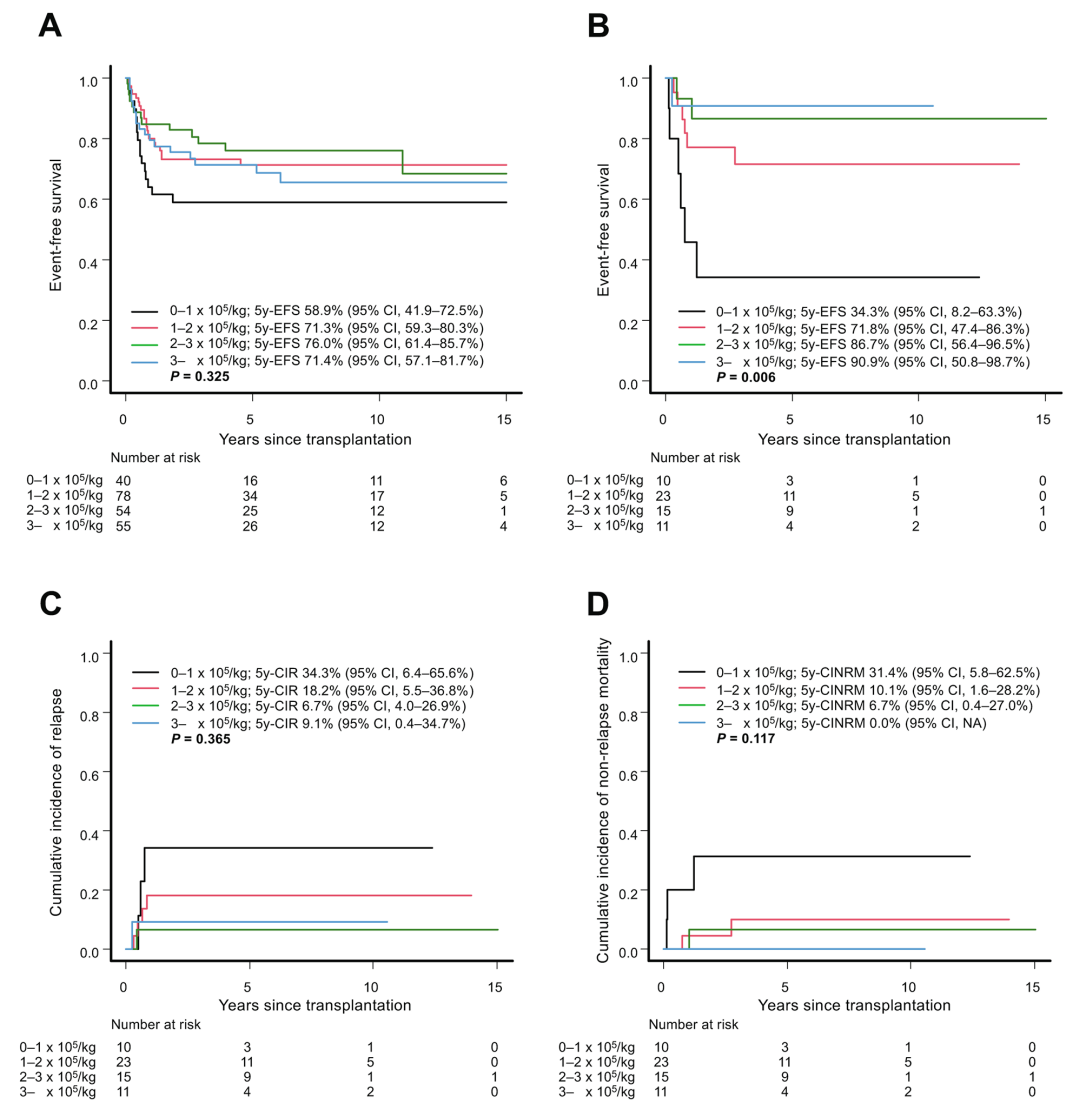
Event-free survival according to the infused CD34 + cell dose in the (**A**) KIR-ligand match group and (**B**) KIR-ligand mismatch group. The cumulative incidence of (**C**) relapse and (**D**) non-relapse mortality according to the infused CD34 + cell dose in the KIR-ligand mismatch group is also shown. EFS, event-free survival; CIR, cumulative incidence of relapse; CINRM, cumulative incidence of non-relapse mortality; CI, confidence interval; NA, not available; KIR, killer immunoglobulin-like receptor

As a number of previous studies have suggested that CD34 + cell doses have an impact on the survival and/or engraftment [9–11], we stratified patients by CD34 + cell dose, and univariate analysis revealed a significant correlation between higher CD34 + cell dose and better EFS in the KIR-L mismatch group, as 5y-EFS was 34.3% for those with less than 1 × 10^5^/kg, 71.8% for those with 1–2 × 10^5^/kg, 86.7% for those with 2–3 × 10^5^/kg, and 90.9% for those with ≥ 3 × 10^5^/kg CD34 + cell doses (Fig. 1B, *p* = 0.006). On the other hand, this correlation was not detected in the KIR-L match group (Fig. 1A; *p* = 0.325). The impacts of CD34 + cell doses on EFS in the KIR-L mismatch group was attributed not only to the lower NRM but also to the lower relapse rate among those receiving higher doses, although neither was significant according to univariate analysis (Fig. 1C and D). These results were similar when we classified the patients into two groups: one with CD34 + cell doses less than the median (referred to as CD34^low^) and the other with CD34 + cell doses equal to or greater than the median (CD34^high^) (Additional file 1: Figure S5).

In the multivariate analysis for EFS, the KIR-L^mismatch^–CD34^high^ (≥ median dose) subgroup was identified as an independent favorable prognostic factor (hazard ratio = 0.19, *p* = 0.029; Table 1). We also performed multivariate analysis for the incidence of relapse, and the KIR-L^mismatch^–CD34^high^ subgroup was identified as an independent favorable prognostic factor (hazard ratio = 0.09, *p* = 0.021; Additional file 1: Table S4).

## Discussion

In this study, CD34 + cell doses were associated with outcomes in the KIR-L mismatch group but not in the KIR-L match group. Consequently, children who received KIR-L-mismatched CBT with high CD34 + cell doses had the best outcome.

To our knowledge, this was the first study in which higher CD34 + cell doses were associated with not only the lower NRM but also the lower relapse rate in the setting of KIR-L-mismatched CBT for children with AML. One interesting study showed that higher infused CD34 + cell doses promoted early reconstitution of NK cells. This, in turn, was associated with a reduced relapse rate and improved survival [12]. These results are compatible with our finding that infusion of higher CD34 + cell doses is important when we expect relapse-reducing effects from NK cells. Moreover, the association between cell dose and survival was strengthened, as we observed a dose-response relationship, as shown in Fig. 1B. Notably, there was no clear association between CD34 + cell dose and survival among those receiving KIR-L-matched CBT (Fig. 1A). This was thought to be a reasonable result, as in the setting of KIR-L-matched CBT, we could not expect a GVL effect from NK cells even when the number of NK cells was greater. As the number of patients in the KIR-L match group was limited (*n* = 61), a clinical trial including a larger number of patients is warranted to verify the results of this study.

### Electronic supplementary material

Below is the link to the electronic supplementary material.

Supplementary Material 1

## Data Availability

The data of this study are not publicly available due to ethical restrictions that it exceeds the scope of the recipient/donor’s consent for research use in the registry.

## Abbreviations

AML: Acute myeloid leukemia
HSCT: Hematopoietic stem cell transplantation
CBT: Cord blood cell transplantation
KIR-L: Killer immunoglobulin-like receptors ligand
NK: Natural killer
GVL: Graft-versus-leukemia
GVHD: Graft-versus-host disease
5y-EFS: 5-year event-free survival
NRM: Non-relapse mortality

## References

[1] Zwaan CM, Kolb EA, Reinhardt D, Abrahamsson J, Adachi S, Aplenc R (2015). Collaborative efforts driving Progress in Pediatric Acute myeloid leukemia. J Clin Oncol.

[2] Eapen M, Rubinstein P, Zhang MJ, Stevens C, Kurtzberg J, Scaradavou A (2007). Outcomes of transplantation of unrelated donor umbilical cord blood and bone marrow in children with acute leukaemia: a comparison study. Lancet.

[3] Milano F, Gooley T, Wood B, Woolfrey A, Flowers ME, Doney K (2016). Cord-blood transplantation in patients with minimal residual disease. N Engl J Med.

[4] Keating AK, Langenhorst J, Wagner JE, Page KM, Veys P, Wynn RF (2019). The influence of stem cell source on transplant outcomes for pediatric patients with acute myeloid leukemia. Blood Adv.

[5] Willemze R, Rodrigues CA, Labopin M, Sanz G, Michel G, Socié G (2009). KIR-ligand incompatibility in the graft-versus-host direction improves outcomes after umbilical cord blood transplantation for acute leukemia. Leukemia.

[6] Yokoyama H, Kanda J, Kawahara Y, Uchida N, Tanaka M, Takahashi S (2021). Reduced leukemia relapse through cytomegalovirus reactivation in killer cell immunoglobulin-like receptor-ligand-mismatched cord blood transplantation. Bone Marrow Transpl.

[7] Davies SM, Iannone R, Alonzo TA, Wang Y-C, Gerbing R, Soni S (2020). A phase 2 trial of KIR-Mismatched unrelated Donor Transplantation using in vivo T cell depletion with Antithymocyte Globulin in Acute Myelogenous Leukemia: children’s Oncology Group AAML05P1 Study. Biol Blood Marrow Transplant.

[8] Verneris MR, Miller JS, Hsu KC, Wang T, Sees JA, Paczesny S (2020). Investigation of donor KIR content and matching in children undergoing hematopoietic cell transplantation for acute leukemia. Blood Adv.

[9] Wagner JE, Barker JN, DeFor TE, Baker KS, Blazar BR, Eide C (2002). Transplantation of unrelated donor umbilical cord blood in 102 patients with malignant and nonmalignant diseases: influence of CD34 cell dose and HLA disparity on treatment-related mortality and survival. Blood.

[10] Konuma T, Kato S, Oiwa-Monna M, Tanoue S, Ogawa M, Isobe M (2017). Cryopreserved CD34 + cell dose, but not total nucleated cell dose, influences hematopoietic recovery and extensive chronic graft-versus-host disease after single-unit cord blood transplantation in adult patients. Biol Blood Marrow Transplant.

[11] Nakasone H, Tabuchi K, Uchida N, Ohno Y, Matsuhashi Y, Takahashi S (2019). Which is more important for the selection of cord blood units for haematopoietic cell transplantation: the number of CD34-positive cells or total nucleated cells?. Br J Haematol.

[12] Zhao F, Shi Y, Chen X, Zhang R, Pang A, Zhai W (2022). Higher dose of CD34 + cells promotes early reconstitution of natural killer cells and is Associated with Better outcomes after unmanipulated hematopoietic stem cell transplantation for myeloid malignancies. Transplantation Cell Therapy.

