# Supplementary material for: A higher CD34 + cell dose correlates with better event-free survival after KIR-ligand mismatched cord blood transplantation for childhood acute myeloid leukemia
Source: J Hematol Oncol. 2024 Apr 29;17:24. doi: 10.1186/s13045-024-01548-3 (PMC11057148; doi:10.1186/s13045-024-01548-3)
Supplement: Supplementary file 1 — Supplementary Material 1 [file 13045_2024_1548_MOESM1_ESM.docx]

**Supplementary Materials**

**A higher CD34+ cell dose correlates with better event-free survival after KIR-ligand mismatched cord blood transplantation for childhood acute myeloid leukemia**

Hisashi Ishida ^1^, Yuta Kawahara ^2^, Daisuke Tomizawa ^3^, Yasuhiro Okamoto ^4^, Asahito Hama ^5^, Yuko Cho ^6^, Katsuyoshi Koh ^7^, Yuhki Koga ^8^, Nao Yoshida ^5^, Maho Sato ^9^, Kiminori Terui ^10^, Naoyuki Miyagawa ^11^, Akihiro Watanabe ^12^, Junko Takita ^13^, Ryoji Kobayashi ^14^, Masaki Yamamoto ^15^, Kenichiro Watanabe ^16^, Keiko Okada ^17^, Koji Kato ^18^, Kimikazu Matsumoto ^3^, Moeko Hino ^19^, Ken Tabuchi ^20^, and Hirotoshi Sakaguchi ^3^

^1^ Department of Pediatrics, Okayama University Hospital, Okayama, Japan

^2^ Department of Pediatrics, Jichi Medical University School of Medicine, Shimotsuke, Japan

^3^ Children's Cancer Center, National Center for Child Health and Development, Tokyo, Japan

^4^ Department of Pediatrics, Graduate School of Medical and Dental Sciences, Kagoshima University, Kagoshima, Japan

^5^ Department of Hematology and Oncology, Children's Medical Center, Japanese Red Cross Aichi Medical Center Nagoya First Hospital, Nagoya, Japan

^6^ Department of Pediatrics, Hokkaido University Hospital, Sapporo, Japan

^7^ Department of Hematology/Oncology, Saitama Children's Medical Center, Saitama, Japan

^8^ Department of Perinatal and Pediatric Medicine, Graduate School of Medical Sciences, Kyushu University, Fukuoka, Japan

^9^ Department of Hematology/Oncology, Osaka Women’s and Children’s Hospital, Izumi, Japan

^10^ Department of Pediatrics, Hirosaki University Hospital, Hirosaki, Japan

^11^ Division of Hematology/Oncology, Kanagawa Children's Medical Center, Yokohama, Japan

^12^ Department of Pediatrics, Niigata Cancer Center Hospital, Niigata, Japan

^13^ Department of Pediatrics, Kyoto University Hospital, Kyoto, Japan

^14^ Department of Hematology/Oncology for Children and Adolescents, Sapporo Hokuyu Hospital, Sapporo, Japan

^15^ Department of Pediatrics, Sapporo Medical University Hospital, Sapporo, Japan

^16^ Department of Hematology and Oncology, Shizuoka Children’s Hospital, Shizuoka, Japan

^17^ Department of Pediatric Hematology/Oncology, Osaka City General Hospital, Osaka, Japan

^18^ Central Japan Cord Blood Bank, Seto, Japan

^19^ Department of Pediatrics, Chiba University School of Medicine, Chiba, Japan

^20^ Japanese Data Center for Hematopoietic Cell Transplantation, Nagakute, Japan

**Supplementary Methods**

***Patients***

The data were collected from the Transplant Registry Unified Management Program (TRUMP), which is sponsored by the Japanese Society for Transplantation and Cellular Therapy and the Japanese Data Center for Hematopoietic Cell Transplantation [1]. The patients were selected according to the following criteria: (1) *de novo* acute myeloid leukemia (AML) other than acute promyelocytic leukemia, (2) age < 16 years and a complete remission (CR)1 or CR2 at the time of receiving hematopoietic stem cell transplantation (HSCT), (3) no prior HSCT, and (4) underwent HSCT between 2000 and 2021. Patients who lacked information on survival or disease recurrence and those with Down syndrome were excluded (**Supplementary Figure S1**). The performance status was assessed as defined by the Eastern Cooperative Oncology Group [2]. KIR-L mismatch in the graft-versus-host direction was defined as lacking a donor KIR-L group (HLA-C1, C2, Bw4, or A3/A11) in a recipient as described previously [3,4]. A myeloablative conditioning (MAC) regimen was defined as total body irradiation (TBI) > 8 Gy, melphalan > 140 mg/m^2^, or busulfan ≥ 9 mg/kg. All the other regimens were analyzed as reduced-intensity conditioning (RIC) regimens [5]. The diagnosis and clinical grading of acute and chronic graft-versus-host disease (GVHD) were performed according to established criteria [6,7]. Adverse cytogenetics were defined as previously described [8]. The patients or their parents provided written consent to undergo transplantation and the use of medical records for research in accordance with the Declaration of Helsinki. The study was approved by the Data Management Committee of the TRUMP and the institutional ethics committee of Okayama University (2305-004).

***Statistical analysis***

The primary endpoint was event-free survival (EFS), and the secondary endpoints were overall survival (OS), cumulative incidence of relapse, and cumulative incidence of non-relapse mortality (NRM) for this study. Probabilities of OS and EFS were calculated using Kaplan–Meier estimators. EFS was defined as survival in continuous CR after HSCT. Relapse was defined as clinical or hematological recurrence of leukemia. NRM was defined as death from any cause other than relapse. Competing events were death without engraftment for neutrophil engraftment, death without relapse for hematological relapse, hematological relapse for NRM, and death without GVHD or hematological relapse for GVHD. Neutrophil engraftment was defined as an absolute neutrophil count ≥ 0.5 x 10^9^/L for 3 consecutive days, and platelet engraftment was defined as a platelet count ≥ 20 x 10^9^/L without transfusion support. Fisher’s exact test and the Kruskal–Wallis test were used to compare categorical and continuous variables, respectively. Univariate analysis was performed using the log-rank test. A multivariate analysis for EFS was conducted using the Cox proportional hazard regression model, and a multivariate analysis for incidence of relapse was conducted using the Fine and Gray competing risk regression model. Measures of association are expressed as hazard ratios (HRs) with 95% confidence intervals (CIs). Along with KIR-L match status, which was of interest in this study, factors that known to affect the outcome of patients after HSCT were entered into the multivariate analysis; these included age at HSCT [9], conditioning regimens [10], year of HSCT [11], remission status (CR1 or CR2) [12], cytogenetic risk [13], cytomegalovirus (CMV) serostatus [14], and the occurrence of acute GVHD [15]. CD34+ cell doses were also entered based on the univariate analysis results. In the multivariate analysis, the occurrence of acute GVHD was treated as a time-dependent variable. For missing data, a complete case analysis was performed. All the statistical analyses were performed using EZR (version 1.54. Saitama Medical Center, Jichi Medical University, Saitama, Japan), a graphical user interface for R (version 4.2.2. The R Foundation for Statistical Computing, Vienna, Austria) [16]. *P* < 0.05 was considered indicative of statistical significance for all analyses.

**Supplementary Figure S1**.

The flow chart and number of patients included in this study.

AML, acute myeloid leukemia; HSCT, hematopoietic stem cell transplantation; CR, complete remission; NA, not available; CBT, cord blood transplantation; BM, bone marrow; PB, peripheral blood; DS, Down syndrome; APL, acute promyelocytic leukemia.

**Supplementary Figure S2**.

Cumulative incidence of (A) neutrophil engraftent and (B) platelet engraftment according to KIR-L match status.

KIR, killer immunoglobulin-like receptors; KIR-L, KIR-ligand; CI, confidence interval.

**Supplementary Figure S3**.

Cumulative incidence of (A) grade II–IV acute GVHD and (B) grade III–IV acute GVHD according to KIR-L match status.

GVHD, graft-versus-host disease; KIR, killer immunoglobulin-like receptors; ; KIR-L, KIR-ligand; CI, confidence interval.

**Supplementary Figure S4**.

Cumulative incidence of (A) chronic GVHD and (B) extensive chronic GVHD according to KIR-L match status.

GVHD, graft-versus-host disease; KIR, killer immunoglobulin-like receptors; ; KIR-L, KIR-ligand; CI, confidence interval.

**Supplementary Figure S5**.

Event-free survival according to the infused CD34+ cell dose in the (A) KIR-ligand match group and (B) KIR-ligand mismatch group. Patients were classified into to groups; one with CD34+ cell doses less than the median (referred to as CD34^low^) and the other with CD34+ cell doses equal to or greater than the median (CD34^high^).

EFS, event-free survival; CI, confidence interval; KIR, killer immunoglobulin-like receptors.

**Supplementary Table S1.** Clinical characteristics of the included patients.

|  |  | **KIR-L** | |  |
| --- | --- | --- | --- | --- |
|  |  | match | mismatch | *P* value |
|  |  | n = 238 | n = 61 |  |
| **Median age, years old [range]** |  | 5.00 [0.00, 15.00] | 4.00 [0.00, 15.00] | 0.985 |
| **Age at HSCT, years old** | 0–4 | 113 (47.5%) | 31 (50.8%) | 0.835 |
|  | 5–9 | 64 (26.9%) | 14 (23.0%) |  |
|  | 10–15 | 61 (25.6%) | 16 (26.2%) |  |
| **TNC*** | < median | 118 (49.6%) | 29 (47.5%) | 0.709 |
|  | ≥ median | 115 (48.3%) | 32 (52.5%) |  |
|  | NA | 5 (2.1%) | 0 (0.0%) |  |
| **CD34+ cells*** | < median | 112 (47.1%) | 31 (50.8%) | 0.852 |
|  | ≥ median | 115 (48.3%) | 28 (45.9%) |  |
|  | NA | 11 (4.6%) | 2 (3.3%) |  |
| **CR status at HSCT** | CR1 | 171 (71.8%) | 41 (67.2%) | 0.528 |
|  | CR2 | 67 (28.2%) | 20 (32.8%) |  |
| **FAB classification** | M0 | 15 (6.3%) | 2 (3.3%) | NA |
|  | M1 | 23 (9.7%) | 2 (3.3%) |  |
|  | M2 | 52 (21.8%) | 14 (23.0%) |  |
|  | M4 | 28 (11.8%) | 5 (8.2%) |  |
|  | M5 | 43 (18.1%) | 9 (14.8%) |  |
|  | M6 | 7 (2.9%) | 2 (3.3%) |  |
|  | M7 | 33 (13.9%) | 11 (18.0%) |  |
|  | Others | 8 (3.4%) | 2 (3.3%) |  |
|  | NA | 29 (12.2%) | 14 (23.0%) |  |
| **HSCT Year** | 2000–2009 | 88 (37.0%) | 9 (14.8%) | 0.001 |
|  | 2010–2021 | 150 (63.0%) | 52 (85.2%) |  |
| **HCT-CI** | 0 | 169 (71.0%) | 50 (82.0%) | 0.015 |
|  | 1 | 14 (5.9%) | 6 (9.8%) |  |
|  | 2 | 3 (1.3%) | 0 (0.0%) |  |
|  | 3 | 0 (0.0%) | 1 (1.6%) |  |
|  | 6 | 1 (0.4%) | 0 (0.0%) |  |
|  | NA | 51 (21.4%) | 4 (6.6%) |  |
| **Conditioning** | BU-based MAC | 12 (5.0%) | 1 (1.6%) | 0.665 |
|  | other non-TBI MAC | 81 (34.0%) | 27 (44.3%) |  |
|  | TBI/CY MAC | 75 (31.5%) | 16 (26.2%) |  |
|  | other TBI MAC | 30 (12.6%) | 7 (11.5%) |  |
|  | FluBasedRIC | 37 (15.5%) | 10 (16.4%) |  |
|  | other RIC | 3 (1.3%) | 0 (0.0%) |  |
| **GVHD prophylaxis** | CSA-based | 74 (31.1%) | 12 (19.7%) | 0.131 |
|  | TAC-based | 159 (66.8%) | 49 (80.3%) |  |
|  | NA | 5 (2.1%) | 0 (0.0%) |  |
| **ATG** | No | 232 (97.5%) | 56 (91.8%) | 0.051 |
|  | Yes | 6 (2.5%) | 5 (8.2%) |  |
| **ECOG PS** | 0–1 | 209 (87.8%) | 53 (86.9%) | 0.200 |
|  | 2–4 | 8 (3.4%) | 5 (8.2%) |  |
|  | NA | 21 (8.8%) | 3 (4.9%) |  |
| **Recipient CMV serostatus** | Negative | 79 (33.2%) | 16 (26.2%) | 0.321 |
|  | Positive | 134 (56.3%) | 41 (67.2%) |  |
|  | NA | 25 (10.5%) | 4 (6.6%) |  |
| **HLA disparities** | 0 | 52 (21.8%) | 0 (0.0%) | <0.001 |
|  | 1 | 101 (42.4%) | 13 (21.3%) |  |
|  | 2 or more | 85 (35.7%) | 48 (78.7%) |  |
| **Donor recipient sex mismatch** | Match | 87 (36.6%) | 28 (45.9%) | 0.556 |
|  | F to M | 63 (26.5%) | 12 (19.7%) |  |
|  | M to F | 53 (22.3%) | 12 (19.7%) |  |
|  | NA | 35 (14.7%) | 9 (14.8%) |  |
| **Cytogenetic risk** | Favorable | 36 (15.1%) | 15 (24.6%) | 0.218 |
|  | Intermediate | 156 (65.5%) | 36 (59.0%) |  |
|  | Adverse | 46 (19.3%) | 10 (16.4%) |  |

*The median total nucleated cell and CD34+ cell doses were 6.7 × 10^7^/kg (range, 0.01–12.3) and 1.9 × 10^5^/kg (range, 0.01–59.4), respectively.

**Abbreviations**: HSCT, hematopoietic stem cell transplantation; TNC, total nucleated cell count; CR, complete remission; FAB, French American British; TBI, total body irradiation; CY, cyclophosphamide; MAC, myeloablative conditioning; HCT-CI, hematopoietic cell transplantation-specific comorbidity index; BU, busulfan; RIC, reduced-intensity conditioning; GVHD, graft-versus-host disease; CSA, cyclosporine A; TAC, tacrolimus; ATG, antithymocyte globulin; ECOG, Eastern Cooperative Oncology Group; PS, performance status; CMV, cytomegalovirus; F, female; M, male; KIR, killer cell immunoglobulin-like receptor; NA, not available

**Supplementary Table S2**. Univariate analysis for overall survival, cumulative incidence of relapse/ non-relapse mortality

|  |  | **n** | **5y OS (95% CI)** | ***P* value** | **5y CIR (95% CI)** | ***P* value** | **5y CINRM (95% CI)** | ***P* value** |
| --- | --- | --- | --- | --- | --- | --- | --- | --- |
| **Age at HSCT, years old** | 0–4 | 144 | 77.9 (69.6–84.1) | 0.782 | 21.6 (15.2–28.8) | 0.835 | 5.1 (2.2–9.6) | 0.405 |
|  | 5–9 | 78 | 71.0 (58.5–80.4) |  | 19.9 (11.7–29.7) |  | 11.9 (5.5–21.1) |  |
|  | 10–15 | 77 | 71.1 (58.8–80.4) |  | 20.5 (12.1–30.5) |  | 11.3 (5.2–20.0) |  |
| **TNC** | < median | 147 | 71.8 (63.3–78.7) | 0.794 | 19.1 (13.1–26.0) | 0.397 | 11.1 (6.4–17.1) | 0.472 |
|  | ≥ median | 147 | 76.1 (67.5–82.6) |  | 22.5 (16.0–29.7) |  | 6.2 (2.9–11.4) |  |
| **CD34+ cells** | < median | 143 | 69.2 (60.1–76.6) | 0.206 | 24.9 (17.9–32.4) | 0.076 | 10.0 (5.6–16.1) | 0.929 |
|  | ≥ median | 143 | 79.2 (71.1–85.3) |  | 15.9 (10.4–22.6) |  | 7.7 (3.9–13.2) |  |
| **KIR-L** | match | 238 | 73.9 (67.4–79.3) | 0.728 | 22.3 (17.1–27.9) | 0.257 | 7.9 (4.8–12.1) | 0.605 |
|  | mismatch | 61 | 76.0 (62.1–85.4) |  | 15.2 (7.4–25.6) |  | 10.8 (4.3–20.7) |  |
| **KIR-L and CD34** | KIR-L match-CD34 low | 112 | 70.5 (60.2–78.6) | 0.247 | 25.0 (17.3–33.5) | 0.198 | 7.9 (3.6–14.4) | 0.315 |
|  | KIR-L mismatch-CD34 low | 31 | 63.6 (42.6–78.7) |  | 24.3 (105–41.1) |  | 17.7 (6.2–34.1) |  |
|  | KIR-L match-CD34 high | 115 | 77.2 (67.8–84.1) |  | 18.1 (11.5–25.8) |  | 8.6 (4.2–15.0) |  |
|  | KIR-L mismatch-CD34 high | 28 | 88.6 (68.6–96.2) |  | 7.1 (1.2–20.7) |  | 3.7 (0.3–16.3) |  |
| **CR status at HSCT** | CR1 | 212 | 77.1 (70.4–82.6) | 0.109 | 19.3 (14.1–25.0) | 0.355 | 7.4 (4.2–11.7) | 0.584 |
|  | CR2 | 87 | 67.6 (55.9–76.8) |  | 24.8 (16.1–34.5) |  | 11.3 (5.5–19.4) |  |
| **HSCT Year** | 2000–2009 | 97 | 69.5 (59.1–77.7) | 0.268 | 21.8 (14.2–30.6) | 0.681 | 11.5 (6.1–18.9) | 0.571 |
|  | 2010–2021 | 202 | 77.1 (70.0–82.7) |  | 20.5 (15.1–26.6) |  | 7.1 (3.8–11.8) |  |
| **HCT-CI** | 0 | 219 | 76.2 (69.4–81.7) | 0.483 | 19.9 (14.8–25.5) | NA | 7.2 (4.1–11.5) | NA |
|  | 1 | 20 | 62.3 (34.4–81.1) |  | 24.9 (7.2–47.9) |  | 18.9 (4.2–41.8) |  |
|  | 2 | 3 | 100.0 (NA) |  | NA |  | NA |  |
|  | 3 | 1 | NA |  | NA |  | NA |  |
|  | 6 | 1 | NA |  | NA |  | NA |  |
| **Conditioning regimen** | chemo-MAC | 120 | 83.0 (74.2–89.0) | < 0.001 | 16.4 (10.3–23.8) | 0.143 | 4.3 (1.6–9.1) | 0.024 |
|  | TBI-MAC | 129 | 61.1 (51.7–69.2) |  | 26.3 (18.9–34.3) |  | 15.1 (9.3–22.1) |  |
|  | RIC | 50 | 90.7 (76.9–96.5) |  | 17.6 (8.1–30.1) |  | 0.0 (0.0–0.0) |  |
| **GVHD prophylaxis** | CSA-based | 86 | 70.7 (59.5–79.3) | 0.284 | 20.4 (12.5–29.6) | 0.944 | 12.2 (6.2–20.3) | 0.103 |
|  | TAC-based | 208 | 75.2 (68.1–80.9) |  | 21.0 (15.6–26.9) |  | 7.3 (4.0–11.8) |  |
| **ATG** | No | 288 | 74.3 (68.4–79.2) | 0.686 | 20.6 (16.0–25.5) | 0.505 | 8.8 (5.7–12.7) | 0.345 |
|  | Yes | 11 | 71.1 (23.3–92.3) |  | 30.0 (6.2–59.3) |  | 0.0 (0.0–0.0) |  |
| **ECOG PS** | 0–1 | 262 | 74.6 (68.4–79.8) | 0.605 | 20.7 (15.9–26.0) | 0.578 | 8.1 (5.0–12.1) | 0.312 |
|  | 2–4 | 13 | 83.3 (48.2–95.6) |  | 25.0 (5.4–51.7) |  | 0.0 (0.0–0.0) |  |
| **Recipient CMV serostatus** | Negative | 95 | 77.9 (67.0–85.6) | 0.564 | 18.5 (11.3–27.1) | 0.530 | 8.6 (3.7–16.1) | 0.512 |
|  | Positive | 175 | 73.6 (65.9–79.8) |  | 22.2 (16.2–28.8) |  | 7.5 (4.1–12.3) |  |
| **Donor recipient sex mismatch** | Match | 115 | 78.8 (69.2–85.8) | 0.297 | 17.5 (11.0–25.3) | 0.322 | 8.2 (3.7–14.8) | 0.567 |
|  | F to M | 75 | 74.2 (62.1–83.0) |  | 27.3 (17.6–37.8) |  | 5.7 (1.8–12.9) |  |
|  | M to F | 65 | 65.5 (50.8–76.8) |  | 21.4 (12.0–32.7) |  | 10.5 (4.2–20.2) |  |
| **Cytogenetic risk** | Favorable | 51 | 79.2 (64.7–88.3) | 0.472 | 17.9 (8.7–29.7) | 0.653 | 8.1 (2.5–17.9) | 0.846 |
|  | Intermediate | 192 | 72.6 (65.2–78.8) |  | 20.6 (15.1–26.7) |  | 9.4 (5.6–14.4) |  |
|  | Adverse | 56 | 75.5 (60.8–85.3) |  | 24.7 (14.0–37.0) |  | 5.6 (1.4–14.1) |  |

**Abbreviations**; CI, confidence interval; OS, overall survival; CIR, cumulative incidence of relapse; CINRM, cumulative incidence of non-relapse mortality; HSCT, hematopoietic stem cell transplantation; TNC, total nucleated cell count; CR, complete remission; KIR, Killer cell immunoglobulin-like receptor; HCT-CI, hematopoietic cell transplantation-specific comorbidity index; TBI, total body irradiation; MAC, myeloablative conditioning; RIC, reduced-intensity conditioning; GVHD, graft-versus-host disease; CSA, cyclosporine A; TAC, tacrolimus; ECOG, Eastern Cooperative Oncology Group; PS, performance status; CMV, cytomegalovirus; F, female; M, male; NA, not available.

**Supplementary Table S3.** Causes of death according to KIR-ligand match status

|  |  | **KIR-L** | |
| --- | --- | --- | --- |
|  |  | Match (n = 60) | Mismatch (n = 13) |
| Original disease | | 32 (53.3%) | 8 (61.5%) |
| Infection |  | 5 (8.3%) | 0 (0%) |
| Noninfection | | 23 (38.3%) | 5 (38.5%) |
|  |  |  |  |
|  | Graft failure | 3 | 1 |
|  | Acute GVHD | 1 | 1 |
|  | Chronic GVHD | 3 | 0 |
|  | Pulmonary (noninfection) | 3 | 1 |
|  | Renal failure | 1 | 0 |
|  | Sinusoidal obstruction syndrome | 2 | 0 |
|  | Hemorrhage | 1 | 1 |
|  | Secondary malignancy | 1 | 0 |
|  | Others | 7 | 1 |
|  | Unknown | 1 | 0 |

**Abbreviations:** KIR, Killer cell immunoglobulin-like receptor; KIR-L, KIR-ligand; GVHD, graft versus host disease.

**Supplementary Table S4.** Multivariate analysis for the incidence of relapse

| Factor | Hazard ratio (95% CI) | *p* value |
| --- | --- | --- |
| KIR-L match-CD34 low | ref |  |
| KIR-L mismatch-CD34 low | 0.99 (0.44-2.24) | 0.978 |
| KIR-L match-CD34 high | 0.51 (0.26-0.99) | 0.045 |
| KIR-L mismatch-CD34 high | 0.09 (0.01-0.70) | 0.021 |
| Age 0–4 | ref |  |
| Age 5–9 | 0.62 (0.31-1.23) | 0.174 |
| Age 10–15 | 0.53 (0.24-1.18) | 0.119 |
| Chemo-based MAC | ref |  |
| TBI-MAC | 1.86 (0.92-3.77) | 0.085 |
| RIC | 0.79 (0.28-2.19) | 0.650 |
| HSCT year 2000–2009 | ref |  |
| HSCT year 2010–2021 | 1.62 (0.83-3.15) | 0.159 |
| CR1 | ref |  |
| CR2 | 1.31 (0.70-2.45) | 0.401 |
| Favorable cytogenetics | ref |  |
| Intermediate cytogenetics | 1.26 (0.53-3.01) | 0.598 |
| Adverse cytogenetics | 1.36 (0.53-3.47) | 0.519 |
| Recipient CMV negative | ref |  |
| Recipient CMV positive | 1.20 (0.65-2.24) | 0.556 |
| no grade II–IV acute GVHD | ref |  |
| grade II–IV acute GVHD | 1.00 (0.57-1.76) | 0.991 |

**Abbreviations**; CI, confidence interval; KIR, Killer cell immunoglobulin-like receptor; MAC, myeloablative conditioning; TBI, total body irradiation; RIC, reduced-intensity conditioning; HSCT, hematopoietic stem cell transplantation; CR, complete remission; CMV, cytomegalovirus; GVHD, graft-versus-host disease; ref, reference.

**Supplementary discussion**

It was conceivable that patients with KIR-L-mismatched cord blood transplantation (CBT) would experience lower engraftment rates and/or a greater incidence of acute GVHD, as they have greater HLA mismatches than KIR-L-matched patients. However, in the present study, the rate of neutrophil engraftment did not differ between the groups, and the incidences of Grade II–IV acute GVHD, Grade III–IV GVHD, chronic GVHD, and extensive chronic GVHD were similar between the KIR-L match and mismatch groups. The 5y-EFS and 5y-OS were also similar between these two groups, suggesting that the selection of KIR-L-mismatched cord blood grafts was at least a feasible option for children with AML.

As noted, the survival outcomes were similar between the KIR-L match and mismatch groups, confirming the results of previous studies [17–20]. However, it was notable that KIR-L-mismatched CBT with higher CD34+ cell doses was identified as an independent prognostic factor for EFS and cumulative incidence of relapse in the multivariate analysis. To our knowledge, this was the first study in which higher CD34+ cell doses were associated with not only lower NRM but also a lower relapse rate in the setting of KIR-L-mismatched CBT for children with AML. Patient age could affect this result, as generally higher cell doses can be achieved in younger patients; thus, we performed multivariate analysis including patient age, and identified KIR-L-mismatched CBT with high CD34+ cell doses as a favorable prognostic factor. Moreover, KIR-L mismatched CBT with high CD34+ cell doses had the best overall EFS when we focused on each age group (separately analyzed patients aged 0–4, 5–9, and 10–15 years; *data not shown*).

In general, the cord blood unit is selected based on the balance between the degree of HLA mismatch and the cell dose [21], and our results potentially suggested updated principles for selecting the graft. When a potent GVL effect is needed for children with high-risk AML, KIR-L mismatched cord blood grafts can be preferable options but should be selected only when adequate CD34+ cell doses are secured (at least 1 × 10^5^/kg, ideally more than 2 × 10^5^/kg). As the body weight of children is generally less than that of adults, grafts with optimal cell doses could be available more easily for children than for adults. On the other hand, when KIR-L-matched cord blood grafts are selected, CD34+ cell doses are more permissive. Moreover, if clinicians have a choice between KIR-L-matched cord blood and KIR-L-mismatched cord blood both with enough CD34+ cell doses (such as more than 2 × 10^5^/kg), KIR-L-mismatched grafts could be a better choice based on our results. However, these results should be validated with other cohorts.

In the present study, only four patients received mycophenolate mofetil (MMF) as GVHD prophylaxis, and in the KIR-L mismatch group, only one patient received MMF. A previous study suggested that MMF acts as a potent inhibitor of natural killer (NK) cells and that the use of MMF in the GVHD prophylaxis regimen might reduce the graft-versus-leukemia (GVL) effect of NK cells [22]. In fact, the different frequencies of MMF usage might partially explain the discrepancy in the efficacy of KIR-L-mismatched CBT among studies. A study from the Eurocord cohort in which 16% of patients received MMF showed a relapse-reducing effect of KIR-L-mismatched CBT, while a study from the Minnesota cohort in which 78% of patients received MMF did not show significant effects [23,24]. Taken together, the comparatively lower use of MMF (1.3%) might be one of the reasons why the GVL effect of KIR-L-mismatched CBT was demonstrated in this study.

Along with the selection of grafts with KIR-L mismatch and higher CD34+ cell doses, which were identified as a favorable prognostic factor, the use of TBI-based MAC regimens was identified as a poor prognostic factor. Previously, TBI-based MAC regimens were preferred for patients receiving CBT because of the expectation of better engraftment [25]; however, more recently, the feasibility of chemotherapy-based MAC or RIC regimens for patients receiving CBT has been demonstrated [26–31]. Moreover, chemotherapy-based regimens have been shown to have similar or sometimes better outcomes than TBI-based regimens, rendering chemotherapy-based regimens more preferable for children with AML [10,32–34]. Together with our results, these findings suggest that chemotherapy-based MAC or RIC regimens are more suitable than TBI-based MAC as conditioning regimens for CBT in children with AML in remission (**Table 1 and Supplementary Table S2**).

This study has several limitations. First, there is a limitation owing to its retrospective design. The background characteristics were comparable between the KIR-L match and mismatch groups, but patients in the KIR-L mismatch group received CBT more recently, a difference which could affect patient outcomes. Thus, to adjust for factors that could affect the outcome, we included the year of HSCT in the multivariate analysis. In addition, there may have been selection bias in which patients with more high-risk diseases received KIR-L mismatch CBT more frequently than KIR-L match CBT with the expectation of GVL effect, but this approach should work rather in the direction of diminishing the advantage of KIR-L mismatch. As such, we expect that our results would not change considerably due to these potential biases. Second, we could not evaluate the CMV reactivation status due to a lack of data, although this status was reported to impact patient outcomes in KIR-L-mismatched CBT [35]. We investigated the impact of recipient CMV serostatus on patient outcome in each group, and we did not identify a significant association between them (**Table 1**). Investigating this issue would be intriguing in future studies. Third, the current practice in Japan differs from that in other countries in several ways, such as more common usage of cord blood or more common usage of busulfan in the context of chemotherapy-based regimens [36]. In addition, heterogeneity in conditioning regimens and GVHD prophylaxis was noted. Moreover, the details of pretransplantation therapy, including the number of chemotherapy cycles, were not available in our dataset. As such, careful consideration should be given to applying these results to the practices in other countries. Fourth, the number of patients in the KIR-L match group was limited (n = 61), and we could not perform multivariate analysis in the KIR-L mismatch group. A clinical trial including a larger number of patients is warranted to verify the results of this study. Finally, we did not investigate the underlying mechanism behind the association between high CD34+ cell doses and the potent GVL effect. Further exploration of the underlying mechanism is also warranted.

**References**

1. Atsuta Y. Introduction of Transplant Registry Unified Management Program 2 (TRUMP2): scripts for TRUMP data analyses, part I (variables other than HLA-related data). International Journal of Hematology. 2016;103:3–10.

2. Zubrod CG, Schneiderman M, Frei E, Brindley C, Lennard Gold G, Shnider B, et al. Appraisal of methods for the study of chemotherapy of cancer in man: Comparative therapeutic trial of nitrogen mustard and triethylene thiophosphoramide. Jounal of Chronic Disseases. 1960;11:7–33.

3. Leung W. Use of NK cell activity in cure by transplant. Brisith Journal of Haematology. 2011;155:14–29.

4. Kawahara Y, Ishimaru S, Tanaka J, Kako S, Hirayama M, Kanaya M, et al. Impact of KIR-ligand mismatch on pediatric T-cell acute lymphoblastic leukemia in unrelated cord blood transplantation. Transplantation and Cellular Therapy. 2022;28:598.e1-598.e8.

5. Giralt S, Ballen K, Rizzo D, Bacigalupo A, Horowitz M, Pasquini M, et al. Reduced-Intensity Conditioning Regimen Workshop: Defining the Dose Spectrum. Report of a Workshop Convened by the Center for International Blood and Marrow Transplant Research. Biology of Blood and Marrow Transplantation. 2009;15:367–9.

6. Przepiorka D, Weisdorf D, Martin P, Klingemann HG, Beatty P, Hows J, et al. 1994 Consensus Conference on Acute GVHD Grading. Bone Marrow Transplantation. 1995;15:825–8

7. Lee SJ, Vogelsang G, Flowers MED. Chronic graft-versus-host disease. Biology of Blood and Marrow Transplantation. 2003;9:215–33.

8. Creutzig U, van den Heuvel-Eibrink MM, Gibson B, Dworzak MN, Adachi S, de Bont E, et al. Diagnosis and management of acute myeloid leukemia in children and adolescents: recommendations from an international expert panel. Blood. 2012;120:3187–205.

9. Løhmann DJA, Abrahamsson J, Ha S-Y, Jónsson ÓG, Koskenvuo M, Lausen B, et al. Effect of age and body weight on toxicity and survival in pediatric acute myeloid leukemia: results from NOPHO-AML 2004. Haematologica. 2016;101:1359–67.

10. de Berranger E, Cousien A, Petit A, Peffault de Latour R, Galambrun C, Bertrand Y, et al. Impact on long-term OS of conditioning regimen in allogeneic BMT for children with AML in first CR: TBI+CY versus BU+CY: a report from the Société Française de Greffe de Moelle et de Thérapie Cellulaire. Bone Marrow Transplantation. 2014;49:382–8.

11. Gooley TA, Chien JW, Pergam SA, Hingorani S, Sorror ML, Boeckh M, et al. Reduced Mortality after Allogeneic Hematopoietic-Cell Transplantation. New England Journal of Medicine. 2010;363:2091–101.

12. Gassas A, Ishaqi MK, Afzal S, Finkelstein-Shechter T, Dupuis A, Doyle J. A comparison of the outcomes of children with acute myelogenous leukemia in either first or second complete remission (CR1 vs CR2) following allogeneic hematopoietic stem cell transplantation at a single transplant center. Bone Marrow Transplantation. 2008;41:941–5.

13. Alloin A-L, Leverger G, Dalle J-H, Galambrun C, Bertrand Y, Baruchel A, et al. Cytogenetics and outcome of allogeneic transplantation in first remission of acute myeloid leukemia: the French pediatric experience. Bone Marrow Transplantation. 2017;52:516–21.

14. Nikolajeva O, Rocha V, Danby R, Ruggeri A, Volt F, Baudoux E, et al. Umbilical Cord Blood Cytomegalovirus Serostatus Does Not Have an Impact on Outcomes of Umbilical Cord Blood Transplantation for Acute Leukemia. Biology of Blood and Marrow Transplantation. 2017;23:1729–35.

15. Kanda J, Umeda K, Kato K, Murata M, Sugita J, Adachi S, et al. Effect of graft-versus-host disease on outcomes after pediatric single cord blood transplantation. Bone Marrow Transplantation. 2020;55:1430–7.

16. Kanda Y. Investigation of the freely available easy-to-use software ‘EZR’ for medical statistics. Bone Marrow Transplantation. 2013;48:452–8.

17. Tanaka J, Morishima Y, Takahashi Y, Yabe T, Oba K, Takahashi S, et al. Effects of KIR ligand incompatibility on clinical outcomes of umbilical cord blood transplantation without ATG for acute leukemia in complete remission. Blood Cancer Journal. 2013;3:e164–e164.

18. Rocha V, Ruggeri A, Spellman S, Wang T, Sobecks R, Locatelli F, et al. Killer Cell Immunoglobulin-Like Receptor–Ligand Matching and Outcomes after Unrelated Cord Blood Transplantation in Acute Myeloid Leukemia. Biology of Blood and Marrow Transplantation. 2016;22:1284–9.

19. Davies SM, Iannone R, Alonzo TA, Wang Y-C, Gerbing R, Soni S, et al. A Phase 2 Trial of KIR-Mismatched Unrelated Donor Transplantation Using in Vivo T Cell Depletion with Antithymocyte Globulin in Acute Myelogenous Leukemia: Children’s Oncology Group AAML05P1 Study. Biology of Blood and Marrow Transplantation. 2020;26:712–7.

20. Verneris MR, Miller JS, Hsu KC, Wang T, Sees JA, Paczesny S, et al. Investigation of donor KIR content and matching in children undergoing hematopoietic cell transplantation for acute leukemia. Blood Advances. 2020;4:1350–6.

21. Barker JN, Scaradavou A, Stevens CE. Combined effect of total nucleated cell dose and HLA match on transplantation outcome in 1061 cord blood recipients with hematologic malignancies. Blood. 2010;115:1843–9.

22. Ohata K, Espinoza JL, Lu X, Kondo Y, Nakao S. Mycophenolic Acid Inhibits Natural Killer Cell Proliferation and Cytotoxic Function: A Possible Disadvantage of Including Mycophenolate Mofetil in the Graft-Versus-Host Disease Prophylaxis Regimen. Biology of Blood and Marrow Transplantation. 2011;17:205–13.

23. Willemze R, Rodrigues CA, Labopin M, Sanz G, Michel G, Socié G, et al. KIR-ligand incompatibility in the graft-versus-host direction improves outcomes after umbilical cord blood transplantation for acute leukemia. Leukemia. 2009;23:492–500.

24. Brunstein CG, Wagner JE, Weisdorf DJ, Cooley S, Noreen H, Barker JN, et al. Negative effect of KIR alloreactivity in recipients of umbilical cord blood transplant depends on transplantation conditioning intensity. Blood. 2009;113:5628–34.

25. Delaney C, Gutman JA, Appelbaum FR. Cord blood transplantation for haematological malignancies: conditioning regimens, double cord transplant and infectious complications. British Journal of Haematology. 2009;147:207–16.

26. Ballen KK, Spitzer TR, Yeap BY, McAfee S, Dey BR, Attar E, et al. Double Unrelated Reduced-Intensity Umbilical Cord Blood Transplantation in Adults. Biology of Blood and Marrow Transplantation. 2007;13:82–9.

27. Sanz J, Wagner JE, Sanz MA, DeFor T, Montesinos P, Bachanova V, et al. Myeloablative Cord Blood Transplantation in Adults with Acute Leukemia: Comparison of Two Different Transplant Platforms. Biology of Blood and Marrow Transplantation. 2013;19:1725–30.

28. Abedin S, Peres E, Levine JE, Choi S, Yanik G, Couriel DR. Double Umbilical Cord Blood Transplantation after Novel Myeloablative Conditioning Using a Regimen of Fludarabine, Busulfan, and Total Lymphoid Irradiation. Biology of Blood and Marrow Transplantation. 2014;20:2062–6.

29. Ruggeri A, Sanz G, Bittencourt H, Sanz J, Rambaldi A, Volt F, et al. Comparison of outcomes after single or double cord blood transplantation in adults with acute leukemia using different types of myeloablative conditioning regimen, a retrospective study on behalf of Eurocord and the Acute Leukemia Working Party of EBMT. Leukemia. 2014;28:779–86.

30. Yamamoto H, Uchida N, Yuasa M, Kageyama K, Ota H, Kaji D, et al. A Novel Reduced-Toxicity Myeloablative Conditioning Regimen Using Full-Dose Busulfan, Fludarabine, and Melphalan for Single Cord Blood Transplantation Provides Durable Engraftment and Remission in Nonremission Myeloid Malignancies. Biology of Blood and Marrow Transplantation. 2016;22:1844–50.

31. Tang B, Zhu X, Zheng C, Liu H, Hao S, Huang D, et al. Retrospective cohort study comparing the outcomes of intravenous busulfan vs. total-body irradiation after single cord blood transplantation. Bone Marrow Transplantation. 2019;54:1614–24.

32. Ishida H, Adachi S, Hasegawa D, Okamoto Y, Goto H, Inagaki J, et al. Comparison of a fludarabine and melphalan combination-based reduced toxicity conditioning with myeloablative conditioning by radiation and/or busulfan in acute myeloid leukemia in Japanese children and adolescents. Pediatr Blood & Cancer. 2015;62:883–9.

33. Bitan M, He W, Zhang M-J, Abdel-Azim H, Ayas MF, Bielorai B, et al. Transplantation for children with acute myeloid leukemia: a comparison of outcomes with reduced intensity and myeloablative regimens. Blood. 2014;123:1615–20.

34. Ishida H, Kato M, Kudo K, Taga T, Tomizawa D, Miyamura T, et al. Comparison of Outcomes for Pediatric Patients With Acute Myeloid Leukemia in Remission and Undergoing Allogeneic Hematopoietic Cell Transplantation With Myeloablative Conditioning Regimens Based on Either Intravenous Busulfan or Total Body Irradiation: A Report From the Japanese Society for Hematopoietic Cell Transplantation. Biology of Blood and Marrow Transplantation. 2015;21:2141–7.

35. Yokoyama H, Kanda J, Kawahara Y, Uchida N, Tanaka M, Takahashi S, et al. Reduced leukemia relapse through cytomegalovirus reactivation in killer cell immunoglobulin-like receptor-ligand-mismatched cord blood transplantation. Bone Marrow Transplantion. 2021;56:1352–63.

36. Yamamoto H. Single cord blood transplantation in Japan; expanding the possibilities of CBT. International Journal of Hematology. 2019;110:39–49.
